# Whole Genome Sequencing Reveals High Prevalence of Antimicrobial Resistance Genes in *Salmonella* Isolates From Diarrheal Patients in Jinan, China

**DOI:** 10.1155/cjid/5033217

**Published:** 2026-04-18

**Authors:** Chuanmin Ma, Na Li, Lei Wang, Kunyan Li, Ming Liu, Jun Zhou, Hui Liu

**Affiliations:** ^1^ Department of Microbiology, Jinan Center for Disease Control and Prevention, Jinan, China; ^2^ Department of Clinical Medicine, The Second Clinical College of Shandong University, Jinan, China; ^3^ Toxicology Research Center, Shandong Academy of Occupational Health and Occupational Medicine, Shandong First Medical University (Shandong Academy of Medical Sciences), Jinan, China, sdfmu.edu.cn

**Keywords:** antimicrobial resistance genes, molecular epidemiology, multilocus sequence typing (MLST), pan-genome, *Salmonella*, whole genome sequencing (WGS)

## Abstract

**Background:**

The global emergence of multidrug‐resistant (MDR) *Salmonella enterica* poses a significant public health threat, particularly in developing countries where antibiotic consumption remains high. However, genomic data on circulating *Salmonella* strains in Eastern China remain limited.

**Objective:**

To characterize the genomic profiles of antimicrobial resistance (AMR) and molecular epidemiology of *Salmonella* isolates from diarrheal patients in Jinan, China (2017–2020).

**Methods:**

A total of 185 *Salmonella* strains isolated from secondary/tertiary hospital outpatients were subjected to whole genome sequencing (WGS) on the Illumina platform. Bioinformatic analyses included antimicrobial resistance gene (ARG) screening (ResFinder and CARD), multilocus sequence typing (MLST), in silico serotyping (SISTR), and pan‐genome analysis.

**Results:**

All isolates exhibited multidrug resistance, with highest rates to fluoroquinolones (100%), aminoglycosides (99.46%), and nitroimidazoles (99.46%). ARG profiling revealed aac(6′)‐Iaa as the predominant gene (98.9%). MLST identified 33 sequence types (STs), dominated by ST34 (28.65%) and ST11 (24.86%). In silico serotyping further confirmed the predominance of the monophasic *Salmonella Typhimurium* (I 1,4, [5],12:i:‐; ST34) and *Salmonella Enteritidis* (ST11). Pan‐genome analysis defined a core genome of 3376 genes (23.35%) and an accessory genome of 9201 cloud genes (63.64%).

**Conclusion:**

This first WGS‐based surveillance in Jinan highlights the predominance of MDR ST34/ST11 *Salmonella* with an extremely high prevalence (100%) of fluoroquinolone resistance. The integration of genomic and historical phenotypic data underscores the urgent need to revise empirical therapy for diarrheal diseases and implement genomic surveillance in public health practice.

## 1. Introduction

China, as a leading global producer in livestock and poultry farming, exhibits substantial demand within its antibiotic market. Antimicrobial agents are extensively utilized in animal husbandry for disease prevention and growth promotion. According to statistics [1], China’s production of antibiotic active pharmaceutical ingredients reached 196,000 tons in 2018, with commonly used antibiotics including penicillin G sodium, amoxicillin, gentamicin, tetracycline hydrochloride, cephalosporins, and sulfonamides. More recently, national and international surveillance reports have continued to highlight sustained antimicrobial consumption and the accelerating burden of antimicrobial resistance (AMR), underscoring the urgent need for updated genomic surveillance of enteric pathogens such as *Salmonella* [2, 3]. The intensive use of antibiotics has precipitated a surge in resistant bacterial strains, with resistance profiles expanding even to novel antibiotics not yet widely deployed in clinical practice. This trend poses significant challenges to public health security and the sustainable development of the livestock industry [4].

Salmonellosis [5], a zoonotic disease caused by various *Salmonella* serotypes, represents one of the most prevalent foodborne illnesses globally. *Salmonella* exhibits a broad host range, colonizing the intestinal tracts of humans, warm‐blooded animals, and cold‐blooded animals. The pathogen invades the bloodstream by disrupting intestinal epithelial cells, causing systemic infections that manifest as sepsis, bacteremia, diarrhea, and fever [6]. A study conducted in Hanoi, Vietnam, revealed that 92.85% of *Salmonella* isolates from retail meat samples demonstrated resistance to at least one antibiotic, with 64.29% exhibiting multidrug resistance (MDR) [7]. This research also identified diverse antimicrobial resistance genes (ARGs) across different *Salmonella* serotypes, underscoring the potential role of retail meat as a significant exposure route for MDR *Salmonella* in humans. Over recent decades, the consequences of antibiotic misuse have become increasingly apparent due to prolonged antimicrobial application in both human and veterinary medicine. The propensity of *Salmonella* resistance genes for horizontal gene transfer (HGT) among strains continuously amplifies its antibiotic resistance.

Whole genome sequencing (WGS) technology has garnered widespread attention over the past decade owing to its unique advantages in data generation, evolutionary and epidemiological studies, microbiological risk assessment, and outbreak investigations. Scientists leverage WGS to rapidly identify ARGs, gene clusters, and mutations within these genetic elements in foodborne pathogens [8]. This article will provide an in‐depth genomic perspective on ARGs in *Salmonella* and review recent research advancements, utilizing insights derived from WGS. Recent genomic epidemiology studies have further demonstrated the utility of WGS in resolving AMR patterns and transmission dynamics of *Salmonella* across different geographical regions [9, 10], highlighting its indispensable role in modern surveillance frameworks.

## 2. Materials and Methods

### 2.1. Bacterial Isolates and Sample Collection

A cohort of 185 *Salmonella* isolates was subjected to WGS in this study. Strains were prospectively collected from diarrheal outpatients attending secondary and tertiary healthcare facilities in Jinan, China, between 2017 and 2020. Each isolate corresponded to a unique patient, and no duplicate isolates from the same individual were included in the study. Clinical specimens (stool and rectal swabs) were processed for pathogen isolation using standardized microbiological protocols. Following isolation and purification, genomic DNA was extracted from each strain using the TIANamp Bacteria DNA Kit (Tiangen Biotech, China).

### 2.2. Culture Media and Instrumentation

Enrichment cultures utilized SBG Broth (Selenite Brilliant Green Enrichment Broth, Luqiao Technology, Beijing), while selective isolation was performed on CHROMagar *Salmonella* plates (CHROMagar, France). Pure cultures were obtained through subculturing on Brain Heart Infusion (BHI) Agar (Luqiao Technology, Beijing). Species‐level identification was confirmed using the Autof MS1000 MALDI‐TOF Mass Spectrometer (Autobio Diagnostics, China) with the MBT Compass reference database.

### 2.3. Isolation and Identification Protocol

Clinical samples (0.3 mL) were enriched in 9 mL SBG Broth at 36 ± 1°C for 16 h. Enriched cultures were streaked onto CHROMagar *Salmonella* plates and incubated at 36 ± 1°C overnight. Presumptive *Salmonella* colonies exhibiting characteristic purple‐to‐mauve pigmentation were subcultured onto BHI agar. Species identification was performed via MALDI‐TOF MS, where bacterial colonies were combined with matrix solution and analyzed against reference spectral libraries; identification scores ≥ 2.0 confirmed *Salmonella enterica*.

### 2.4. WGS and Bioinformatics Workflow

Isolates were cultured in Tryptic Soy Broth at 37 ± 2°C for 16 ± 1 h prior to DNA extraction. Genomic DNA was purified using the DNeasy Blood & Tissue Kit (Qiagen, Germany), with DNA concentrations quantified using a Qubit 3.0 Fluorometer (Thermo Fisher Scientific, USA). WGS libraries were constructed using the KAPA HyperPrep Kit (Roche, USA). Briefly, 0.2 μg of genomic DNA per sample was fragmented by sonication to ∼350 bp. The fragments were end‐repaired, A‐tailed, and ligated with Illumina indexing adapters. Following a limited‐cycle PCR amplification, libraries were purified using AMPure XP beads (Beckman Coulter, USA). Library quality and size distribution were assessed on an Agilent 5400 system (Agilent Technologies, USA), and quantification was performed by quantitative PCR (qPCR). The pooled libraries were sequenced on an Illumina MiSeq platform (Illumina, USA) to generate 2 × 150 bp paired‐end reads.

Bioinformatic analyses were executed through the TORMES pipeline (v1.3.0). Raw sequences underwent quality trimming with Trimmomatic (v0.39) to remove adapters and low‐quality bases (Phred score < 20). De novo assembly was performed using SPAdes (v3.15) with k‐mer optimization (21–127 bp). Contigs were ordered against the *S. enterica* subsp. *enterica* serovar *Typhimurium* LT2 reference genome (RefSeq NC_003197) using ABACAS. Genome annotation was conducted with Prokka (v1.14.6). Multilocus sequence typing (MLST) employed the *Salmonella* 7‐locus scheme (*aroC*, *dnaN*, *hemD*, *hisD*, *purE*, *sucA*, *thrA*) via MLST v2.0. ARGs were identified using ResFinder (v4.1) and the Comprehensive Antibiotic Resistance Database (CARD v3.2.5), applying thresholds of ≥ 90% sequence identity and ≥ 60% coverage. Pan‐genome analysis was performed with Roary (v3.13) defining core genes as those present in ≥ 99% of isolates and cloud genes as strain‐specific sequences.

The serovar (serotype) of all *Salmonella* isolates was determined in silico from the WGS data using the *Salmonella* In Silico Typing Resource (SISTR) platform (v1.1.2). The analysis was performed by uploading the assembled genome sequences in FASTA format to the public SISTR server. The platform predicts the serovar by rapidly comparing the genomes against a curated database, deriving the antigenic formula (O and H antigens) and final serovar designation based on the White–Kauffmann–Le Minor scheme. Only isolates that passed the internal quality control checks (qc_status: “PASS”) were included in the final serovar distribution analysis.

The following antibiotic abbreviations are used throughout the manuscript: ampicillin (AMP), tetracycline (TET), ciprofloxacin (CIP), chloramphenicol (CHL), streptomycin (STR), trimethoprim–sulfamethoxazole (SXT), nalidixic acid (NAL), ceftazidime (CAZ), and cefotaxime (CTX).

## 3. Results

**FIGURE 1:**
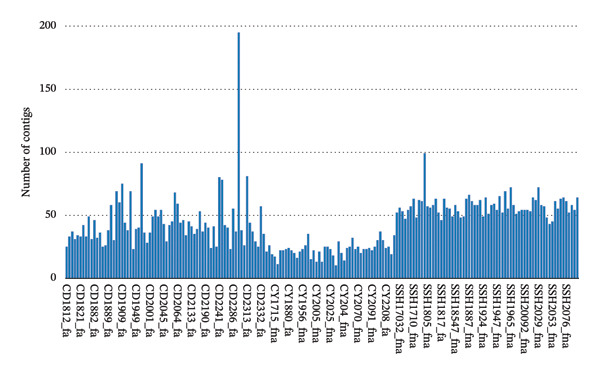
Distribution of contig numbers obtained from de novo assembly of whole genome sequencing data for 185 *Salmonella* isolates. Each bar represents the number of contigs per isolate following quality trimming and SPAdes assembly.

**FIGURE 2:**
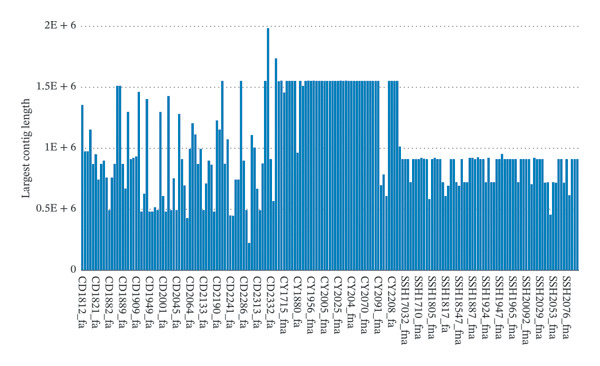
Distribution of maximum contig lengths (bp) generated from de novo genome assembly of 185 *Salmonella* isolates. The plot illustrates the longest contig obtained for each isolate, reflecting assembly continuity and genome integrity.

All 185 clinical isolates were definitively identified as *S. enterica* through concordant MALDI‐TOF mass spectrometry and whole genome sequence–based phylogenetic analysis. High‐quality genomic assemblies were obtained following rigorous quality control and de novo assembly, with isolates exhibiting a mean of 43.78 contigs (Figure 1) and an average maximum contig length of 1,020,573 bp (Figure 2), confirming robust assembly integrity suitable for downstream characterization.

**FIGURE 3:**
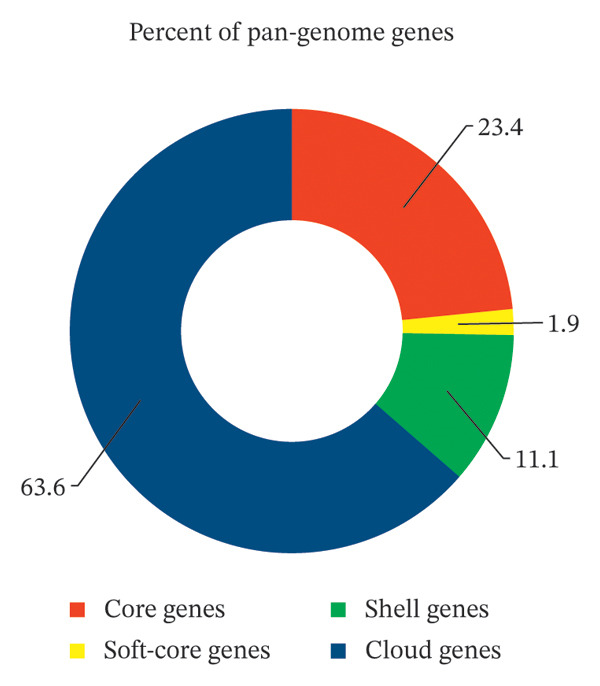
Pan‐genome composition of 185 *Salmonella* isolates based on Roary analysis. The bar chart shows the distribution of core genes (≥ 99% of isolates), shell genes (moderately conserved), and cloud genes (strain‐specific), illustrating the genomic diversity of the population.

Pan‐genome analysis revealed substantial genetic diversity across the collection, with a total gene repertoire of 14,457 genes. The core genome conserved across ≥ 99% of isolates comprised 3376 genes (23.35%), while accessory elements included 9201 strain‐specific cloud genes (63.64%) and 1880 shell genes (13.01%) exhibiting intermediate prevalence (Figure 3). MLST resolved the isolates into 33 distinct sequence types (STs), dominated by the globally disseminated lineages ST34 (53 isolates, 28.65%) and ST11 (46 isolates, 24.86%), which collectively represented over half of the collection (Supporting Table 1).

In silico serotyping of the 185 isolates revealed a diverse population comprising 15 distinct serovars, with a clear dominance of two epidemic lineages (Supporting Table 2). The most prevalent serovar was the monophasic variant of *Salmonella Typhimurium*, designated as I 1,4, [5],12:i:‐, which accounted for 53 isolates (28.65%). This serovar demonstrated a perfect correspondence with ST ST34. The second most common serovar was *Salmonella Enteritidis*, comprising 46 isolates (24.86%), all of which belonged to ST11. Other notable serovars identified included *Salmonella London* (16 isolates, 8.65%), *Salmonella Goldcoast* (7 isolates, 3.78%), and *Salmonella Agona* (6 isolates, 3.24%). The overall serovar distribution showed strong concordance with the MLST‐based phylogeny, reinforcing the link between specific serovars and their underlying genetic lineages.

Alarming resistance profiles were observed among the isolates. Universal resistance to fluoroquinolones (100%) was documented, accompanied by very high resistance to aminoglycosides (99.46%) and nitroimidazoles (99.46%). The identical resistance rates for aminoglycosides and nitroimidazoles resulted from the presence of only one susceptible isolate in each category and likely reflect the predominance of multidrug‐resistant clones within the collection. Substantial resistance rates were also noted for SXT (55.14%), TET (30.81%), and CHL (20.54%).

**FIGURE 4:**
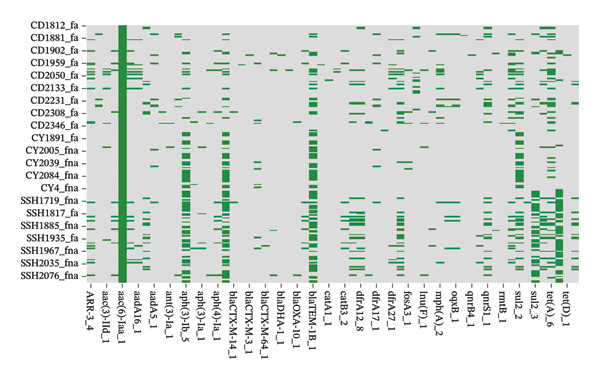
Heatmap of acquired antimicrobial resistance genes identified by ResFinder in 185 *Salmonella* isolates. Rows represent isolates and columns represent resistance genes. Color intensity indicates gene presence or absence, highlighting the distribution of dominant resistance determinants across the collection.

**FIGURE 5:**
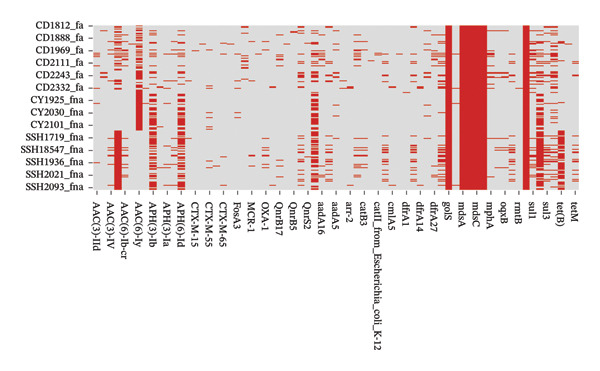
Heatmap of antimicrobial resistance–related genes identified using the CARD database in 185 *Salmonella* isolates. The heatmap displays efflux‐associated genes and other resistance determinants, with color coding indicating gene detection frequency among isolates.

Comprehensive ARG analysis revealed high‐prevalence determinants through complementary databases. ResFinder screening identified dominant acquired resistance genes including the aminoglycoside‐modifying enzymes (AMEs) aac(6′)‐Iaa (98.9%), aph(3″)‐Ib (95.1%), and aph(6)‐Id (92.4%), alongside the extended‐spectrum β‐lactamase (ESBL) gene blaTEM‐1B (87.6%) (Figure 4). CARD analysis demonstrated universal presence of efflux‐associated genes (mdsA, mdsB, and mdsC; 100%) and the glyoxalase resistance gene golS (100%) (Figure 5). Fluoroquinolone resistance mechanisms were multifaceted, involving both efflux pump genes (emrA, emrB, and mdtK; 98.9%–100%) and target mutations in quinolone resistance–determining regions (QRDRs) (GyrA‐S83F in 97.3%; ParC‐S80I in 95.7%).

## 4. Discussion

### 4.1. Current Status of *Salmonella* Antibiotic Resistance in China

Research on antibiotic resistance genes in *Salmonella* has been extensive globally. Mezal et al. [11] isolated 32 strains of *S. enterica* from clinical samples in 2010. Ten of these strains were susceptible to AMP, CHL, gentamicin, kanamycin, NAL, SXT, STR, and TET, while 21 exhibited resistance to AMP and TET, and one was resistant to NAL. Zhou et al. [12] analyzed the antibiotic resistance genes of 146 *Salmonella* isolates from retail chicken products in Shanghai in 2018. They found that approximately 42% of the isolates were susceptible to 13 antibiotics, with the highest resistance to AMP (50.70%), followed by SXT (49.32%) and TET (17.12%). Around 20% of the isolates showed resistance to three or more antibiotics. Additionally, Guo et al. [13] conducted a comprehensive study of *Salmonella* strains isolated from food animals in China from 2015 to 2021, revealing resistance genes related to STR (*aadA*), β‐lactams (*blaTEM, blaCMY, blaSHV,* and *blaCTX*), TET (*tetA* and *tetB*), and sulfonamides (*sulI, sulII,* and *sulIII*).

*Salmonella* exhibits severe resistance to AMP, TET, and fluoroquinolones, with the global prevalence of MDR strains having surged from approximately 20% in the 1990s to about 70% in the current century [14]. This escalating trend poses substantial challenges to the clinical management of infectious diseases and represents a persistent threat to public health. Evidence from multiple geographical regions underscores the widespread nature of MDR *Salmonella*, including reports from Saudi Arabia [15], Belgium [16], and Taiwan [17]. Complementing these findings, a recent WGS study in Saudi Arabia revealed that MDR *Salmonella* isolates from retail chicken frequently carried epidemic plasmids, such as IncFIB and IncA/C2, harboring resistance genes against β‐lactams, TETs, and sulfonamides [18]. Similarly, in China, Guan et al. [19] identified the carbapenem resistance gene blaNDM located on IncFII plasmids within *Salmonella* isolates from waterfowl, highlighting the emergence of resistance to last‐resort antibiotics. These collective findings underscore the transboundary risk of MDR *Salmonella* transmission through diverse reservoirs, with serious implications for both human and animal health. Given the progressive expansion of resistance mechanisms, the current antibiotic resistance profile of *Salmonella* constitutes a pressing public health issue that demands intensified surveillance and intervention strategies.

To contextualize our genotypic findings with local phenotypic resistance patterns, we compiled historical antimicrobial susceptibility testing data for clinically prevalent *Salmonella* serovars in the Jinan region (Supporting Table 3). This dataset reveals established phenotypic resistance profiles that strongly corroborate the resistance genes identified in our current WGS‐based study. For instance, the high prevalence of the tetA gene (95%) and the blaTEM‐1B gene (87.6%) in our isolates is directly reflected in the historical high resistance rates to TET and AMP, respectively, which were observed across a wide range of serovars including Goldcoast, Stanley, London, and Infantis. Similarly, the widespread phenotypic resistance to SXT and STR in the historical data aligns with the common detection of sul and aadA family genes in our genomes. Notably, for fluoroquinolones, while the aac(6′)‐Iaa gene was ubiquitous, the phenotypic resistance to CIP, a commonly used fluoroquinolone, was variable in the historical data and appeared to be serovar‐dependent, suggesting the involvement of additional regulatory mechanisms or the need for specific mutational backgrounds for full resistance expression. The remarkable concordance between the historical phenotypic resistance patterns of circulating serovars and the contemporary resistome uncovered by WGS highlights a strong genotype–phenotype correlation. This consistency suggests that the identified resistance determinants are not only prevalent but are also functionally active and have played a sustained role in shaping the local AMR landscape of *Salmonella*. This integration of historical phenotypic evidence significantly strengthens the validity and public health relevance of our genotypic predictions.

### 4.2. Advances in *Salmonella* Resistance Mechanisms

Bacterial antibiotic resistance can be either intrinsic or acquired. Intrinsic resistance is determined by chromosomal genes, whereas acquired resistance is primarily driven by excessive or repeated exposure to antimicrobial agents. Acquired resistance mechanisms include active efflux systems, enzymatic inactivation of antibiotics, and structural alterations of drug target sites. Common antimicrobial agents used against Gram‐negative enterobacteria include β‐lactams, aminoglycosides, TETs, sulfonamides, and fluoroquinolones.

β‐Lactam antibiotics exert their antibacterial effects by inhibiting bacterial cell wall synthesis through the β‐lactam ring structure. In *Salmonella*, resistance to β‐lactams is primarily mediated by the production of β‐lactamases, which hydrolyze the β‐lactam ring and prevent antibiotic binding to penicillin‐binding proteins (PBPs). ESBLs, encoded by genes located on chromosomal or mobile genetic elements (MGEs) such as plasmids, facilitate HGT between strains. Resistance is commonly associated with blaTEM, blaSHV, blaCTX, blaCMY, and blaOXA genes [20]. Another clinically important β‐lactamase is AmpC, which hydrolyzes cephalosporins and penicillins and contributes to resistance against β‐lactamase inhibitors, posing significant challenges for treatment [21].

Resistance to aminoglycosides in *Salmonella* is mainly attributed to AMEs, including acetyltransferases (AAC), adenyltransferases (ANT), and phosphotransferases (APH). These enzymes chemically modify aminoglycosides, thereby reducing their antibacterial activity. MGEs further promote the horizontal dissemination of these resistance determinants among bacterial populations.

TET resistance is primarily mediated by ribosomal protection proteins (RPPs) encoded by TET resistance genes such as tetM, tetO, and tetS, which protect the ribosome from TET binding and restore protein synthesis [22]. Efflux‐mediated mechanisms may also contribute to decreased intracellular drug accumulation.

Sulfonamides inhibit bacterial folate synthesis by targeting enzymes such as dihydropteroate synthase and dihydrofolate reductase. Resistance in *Salmonella* is mainly mediated by plasmid‐borne sul1, sul2, and sul3 genes, which encode altered target enzymes with reduced affinity for sulfonamides [23].

Fluoroquinolones, a major subclass of quinolones, inhibit bacterial DNA synthesis by targeting DNA gyrase and topoisomerase IV. Resistance to quinolones is largely associated with mutations in the QRDRs of gyrA, gyrB, parC, and parE, which reduce drug‐binding affinity [24]. Additional mechanisms include enzymatic modification by aac(6′)‐Ib‐cr, which particularly affects fluoroquinolones, and active efflux mediated by the AcrAB‐TolC pump system, both of which contribute to multidrug resistance [25].

## 5. Conclusion

In conclusion, this WGS‐based study provides a high‐resolution genomic characterization of *Salmonella* isolates from diarrheal patients in Jinan. We documented a high prevalence of MDR clones, particularly ST34 and ST11, and delineated their comprehensive resistome profiles. The extremely high prevalence of fluoroquinolone resistance (100%), together with very high aminoglycoside resistance rates, as predicted by WGS, poses a significant concern for empirical therapy. This work establishes a critical baseline of genomic data and demonstrates the utility of WGS as a powerful tool for routine surveillance, enabling precise tracking of serovars, STs, and resistance determinants. The findings underscore the urgent need for continued genomic surveillance and prudent antibiotic use in clinical and community settings. Future studies involving experimental validation are warranted to further elucidate the functional and mechanistic aspects of the resistance and virulence genes identified here.

## Author Contributions

**Chuanmin Ma**: conceptualization (lead); formal analysis (lead); investigation (equal); and writing–original draft (lead). **Na Li**: investigation (lead); data curation (lead); formal analysis (equal); and writing–original draft (equal). **Lei Wang**: formal analysis (equal); methodology (equal); and data curation (equal). **Kunyan Li**: methodology (equal); formal analysis (equal); and data curation (equal). **Ming Liu**: conceptualization (equal) and formal analysis (equal). **Jun Zhou**: project administration (lead). **Hui Liu**: supervision (lead); project administration (equal); and funding acquisition (lead).

## Funding

This study was supported by the Science and Technology Program of Jinan Municipal Health Commission (Nos. 2024307006 and 2025206004).

## Ethics Statement

This study was approved by the Ethics Committee of Jinan Center for Disease Control and Prevention (Ethical Approval Number: JNCDC‐2025‐020), and written informed consent was obtained from all the participants.

## Conflicts of Interest

The authors declare no conflicts of interest.

## Supporting Information

Supporting Table S1. Multilocus sequence typing (MLST) profiles of *Salmonella* isolates included in this study.

Supporting Table S2. Serotyping results and antigenic formulas of *Salmonella* isolates.

Supporting Table S3. Historical phenotypic antimicrobial resistance profiles of prevalent *Salmonella* serovars in the Jinan region.

## Supporting information


**Supporting Information** Additional supporting information can be found online in the Supporting Information section.

## Data Availability

All raw data and code are available upon request.
