# Supplementary material for: Whole Genome Sequencing Reveals High Prevalence of Antimicrobial Resistance Genes in Salmonella Isolates From Diarrheal Patients in Jinan, China
Source: Can J Infect Dis Med Microbiol. 2026 Apr 18;2026:5033217. doi: 10.1155/cjid/5033217 (PMC13091231; doi:10.1155/cjid/5033217)
Supplement: Supplementary file 1 — Supporting Information Additional supporting information can be found online in the Supporting Information section. [file CJID-2026-5033217-s001.docx]

**Table S1. Multilocus sequence typing (MLST) profiles of *Salmonella* isolates**

| Sample | Scheme | ST | 1 | 2 | 3 | 4 | 5 | 6 | 7 |
| --- | --- | --- | --- | --- | --- | --- | --- | --- | --- |
| CD1812_fa | senterica | 516 | aroC(84) | dnaN(11) | hemD(16) | hisD(42) | purE(40) | sucA(71) | thrA(4) |
| CD1814_fa | senterica | 463 | aroC(92) | dnaN(125) | hemD(78) | hisD(128) | purE(138) | sucA(9) | thrA(141) |
| CD1815_fa | senterica | 463 | aroC(92) | dnaN(125) | hemD(78) | hisD(128) | purE(138) | sucA(9) | thrA(141) |
| CD1816_fa | senterica | 1959 | aroC(313) | dnaN(445) | hemD(3) | hisD(507) | purE(480) | sucA(388) | thrA(333) |
| CD1819_fa | senterica | 413 | aroC(15) | dnaN(70) | hemD(93) | hisD(78) | purE(113) | sucA(6) | thrA(68) |
| CD1821_fa | senterica | 504 | aroC(157) | dnaN(141) | hemD(17) | hisD(16) | purE(33) | sucA(13) | thrA(4) |
| CD1822_fa | senterica | 2529 | aroC(124) | dnaN(110) | hemD(35) | hisD(204) | purE(95) | sucA(521) | thrA(16) |
| CD1844_fa | senterica | 413 | aroC(15) | dnaN(70) | hemD(93) | hisD(78) | purE(113) | sucA(6) | thrA(68) |
| CD1863_fa | senterica | 13 | aroC(3) | dnaN(3) | hemD(7) | hisD(4) | purE(3) | sucA(3) | thrA(7) |
| CD1881_fa | senterica | 543 | aroC(117) | dnaN(135) | hemD(18) | hisD(12) | purE(162) | sucA(162) | thrA(38) |
| CD1882_fa | senterica | 155 | aroC(10) | dnaN(60) | hemD(58) | hisD(66) | purE(6) | sucA(65) | thrA(16) |
| CD1883_fa | senterica | 543 | aroC(117) | dnaN(135) | hemD(18) | hisD(12) | purE(162) | sucA(162) | thrA(38) |
| CD1884_fa | senterica | 413 | aroC(15) | dnaN(70) | hemD(93) | hisD(78) | purE(113) | sucA(6) | thrA(68) |
| CD1886_fa | senterica | 29 | aroC(16) | dnaN(16) | hemD(20) | hisD(18) | purE(8) | sucA(12) | thrA(18) |
| CD1888_fa | senterica | 29 | aroC(16) | dnaN(16) | hemD(20) | hisD(18) | purE(8) | sucA(12) | thrA(18) |
| CD1889_fa | senterica | 413 | aroC(15) | dnaN(70) | hemD(93) | hisD(78) | purE(113) | sucA(6) | thrA(68) |
| CD1890_fa | senterica | 543 | aroC(117) | dnaN(135) | hemD(18) | hisD(12) | purE(162) | sucA(162) | thrA(38) |
| CD1901_fa | senterica | 26 | aroC(14) | dnaN(13) | hemD(18) | hisD(12) | purE(14) | sucA(18) | thrA(1) |
| CD1902_fa | senterica | 34 | aroC(10) | dnaN(19) | hemD(12) | hisD(9) | purE(5) | sucA(9) | thrA(2) |
| CD1904_fa | senterica | 34 | aroC(10) | dnaN(19) | hemD(12) | hisD(9) | purE(5) | sucA(9) | thrA(2) |
| CD1909_fa | senterica | 34 | aroC(10) | dnaN(19) | hemD(12) | hisD(9) | purE(5) | sucA(9) | thrA(2) |
| CD1918_fa | senterica | 808 | aroC(10) | dnaN(71) | hemD(43) | hisD(12) | purE(190) | sucA(20) | thrA(18) |
| CD1920_fa | senterica | 155 | aroC(10) | dnaN(60) | hemD(58) | hisD(66) | purE(6) | sucA(65) | thrA(16) |
| CD1932_fa | senterica | 469 | aroC(92) | dnaN(107) | hemD(79) | hisD(156) | purE(64) | sucA(151) | thrA(87) |
| CD1939_fa | senterica | 516 | aroC(84) | dnaN(11) | hemD(16) | hisD(42) | purE(40) | sucA(71) | thrA(4) |
| CD1949_fa | senterica | 155 | aroC(10) | dnaN(60) | hemD(58) | hisD(66) | purE(6) | sucA(65) | thrA(16) |
| CD1950_fa | senterica | 155 | aroC(10) | dnaN(60) | hemD(58) | hisD(66) | purE(6) | sucA(65) | thrA(16) |
| CD1959_fa | senterica | 17 | aroC(8) | dnaN(8) | hemD(11) | hisD(11) | purE(5) | sucA(11) | thrA(15) |
| CD1969_fa | senterica | 155 | aroC(10) | dnaN(60) | hemD(58) | hisD(66) | purE(6) | sucA(65) | thrA(16) |
| CD1970_fa | senterica | 26 | aroC(14) | dnaN(13) | hemD(18) | hisD(12) | purE(14) | sucA(18) | thrA(1) |
| CD2001_fa | senterica | 307 | aroC(2) | dnaN(14) | hemD(24) | hisD(14) | purE(2) | sucA(19) | thrA(107) |
| CD2008_fa | senterica | 155 | aroC(10) | dnaN(60) | hemD(58) | hisD(66) | purE(6) | sucA(65) | thrA(16) |
| CD2014_fa | senterica | 241 | aroC(43) | dnaN(47) | hemD(49) | hisD(16) | purE(41) | sucA(15) | thrA(3) |
| CD2018_fa | senterica | 155 | aroC(10) | dnaN(60) | hemD(58) | hisD(66) | purE(6) | sucA(65) | thrA(16) |
| CD2032_fa | senterica | 13 | aroC(3) | dnaN(3) | hemD(7) | hisD(4) | purE(3) | sucA(3) | thrA(7) |
| CD2045_fa | senterica | 155 | aroC(10) | dnaN(60) | hemD(58) | hisD(66) | purE(6) | sucA(65) | thrA(16) |
| CD2050_fa | senterica | 22 | aroC(12) | dnaN(2) | hemD(15) | hisD(14) | purE(11) | sucA(14) | thrA(16) |
| CD2059_fa | senterica | 13 | aroC(3) | dnaN(3) | hemD(7) | hisD(4) | purE(3) | sucA(3) | thrA(7) |
| CD2060_fa | senterica | 214 | aroC(14) | dnaN(72) | hemD(21) | hisD(12) | purE(6) | sucA(19) | thrA(15) |
| CD2061_fa | senterica | 1628 | aroC(46) | dnaN(60) | hemD(10) | hisD(9) | purE(6) | sucA(12) | thrA(17) |
| CD2064_fa | senterica | 40 | aroC(19) | dnaN(20) | hemD(3) | hisD(20) | purE(5) | sucA(22) | thrA(22) |
| CD2105_fa | senterica | 32 | aroC(17) | dnaN(18) | hemD(22) | hisD(17) | purE(5) | sucA(21) | thrA(19) |
| CD2111_fa | senterica | 358 | aroC(5) | dnaN(110) | hemD(35) | hisD(122) | purE(2) | sucA(19) | thrA(22) |
| CD2114_fa | senterica | 413 | aroC(15) | dnaN(70) | hemD(93) | hisD(78) | purE(113) | sucA(6) | thrA(68) |
| CD2122_fa | senterica | 40 | aroC(19) | dnaN(20) | hemD(3) | hisD(20) | purE(5) | sucA(22) | thrA(22) |
| CD2133_fa | senterica | 155 | aroC(10) | dnaN(60) | hemD(58) | hisD(66) | purE(6) | sucA(65) | thrA(16) |
| CD2176_fa | senterica | 469 | aroC(92) | dnaN(107) | hemD(79) | hisD(156) | purE(64) | sucA(151) | thrA(87) |
| CD2184_fa | senterica | 13 | aroC(3) | dnaN(3) | hemD(7) | hisD(4) | purE(3) | sucA(3) | thrA(7) |
| CD2186_fa | senterica | 13 | aroC(3) | dnaN(3) | hemD(7) | hisD(4) | purE(3) | sucA(3) | thrA(7) |
| CD2187_fa | senterica | 155 | aroC(10) | dnaN(60) | hemD(58) | hisD(66) | purE(6) | sucA(65) | thrA(16) |
| CD2190_fa | senterica | 22 | aroC(12) | dnaN(2) | hemD(15) | hisD(14) | purE(11) | sucA(14) | thrA(16) |
| CD2196_fa | senterica | 1959 | aroC(313) | dnaN(445) | hemD(3) | hisD(507) | purE(480) | sucA(388) | thrA(333) |
| CD2207_fa | senterica | 11 | aroC(5) | dnaN(2) | hemD(3) | hisD(7) | purE(6) | sucA(6) | thrA(11) |
| CD2230_fa | senterica | 2529 | aroC(124) | dnaN(110) | hemD(35) | hisD(204) | purE(95) | sucA(521) | thrA(16) |
| CD2231_fa | senterica | 590 | aroC(188) | dnaN(112) | hemD(121) | hisD(14) | purE(176) | sucA(183) | thrA(1) |
| CD2241_fa | senterica | 469 | aroC(92) | dnaN(107) | hemD(79) | hisD(156) | purE(64) | sucA(151) | thrA(87) |
| CD2243_fa | senterica | 469 | aroC(92) | dnaN(107) | hemD(79) | hisD(156) | purE(64) | sucA(151) | thrA(87) |
| CD2254_fa | senterica | 2529 | aroC(124) | dnaN(110) | hemD(35) | hisD(204) | purE(95) | sucA(521) | thrA(16) |
| CD2277_fa | senterica | 2529 | aroC(124) | dnaN(110) | hemD(35) | hisD(204) | purE(95) | sucA(521) | thrA(16) |
| CD2281_fa | senterica | 11 | aroC(5) | dnaN(2) | hemD(3) | hisD(7) | purE(6) | sucA(6) | thrA(11) |
| CD2286_fa | senterica | 13 | aroC(3) | dnaN(3) | hemD(7) | hisD(4) | purE(3) | sucA(3) | thrA(7) |
| CD2287_fa | senterica | 155 | aroC(10) | dnaN(60) | hemD(58) | hisD(66) | purE(6) | sucA(65) | thrA(16) |
| CD2302_fa | senterica | 198 | aroC(76) | dnaN(14) | hemD(3) | hisD(77) | purE(64) | sucA(64) | thrA(67) |
| CD2308_fa | senterica | 358 | aroC(5) | dnaN(110) | hemD(35) | hisD(122) | purE(2) | sucA(19) | thrA(22) |
| CD2310_fa | senterica | 64 | aroC(10) | dnaN(14) | hemD(15) | hisD(31) | purE(25) | sucA(20) | thrA(33) |
| CD2313_fa | senterica | 49 | aroC(5) | dnaN(14) | hemD(21) | hisD(9) | purE(6) | sucA(12) | thrA(17) |
| CD2321_fa | senterica | 155 | aroC(10) | dnaN(60) | hemD(58) | hisD(66) | purE(6) | sucA(65) | thrA(16) |
| CD2324_fa | senterica | 14 | aroC(7) | dnaN(6) | hemD(8) | hisD(8) | purE(7) | sucA(8) | thrA(13) |
| CD2325_fa | senterica | 11 | aroC(5) | dnaN(2) | hemD(3) | hisD(7) | purE(6) | sucA(6) | thrA(11) |
| CD2330_fa | senterica | 50 | aroC(5) | dnaN(21) | hemD(18) | hisD(9) | purE(6) | sucA(12) | thrA(17) |
| CD2332_fa | senterica | 198 | aroC(76) | dnaN(14) | hemD(3) | hisD(77) | purE(64) | sucA(64) | thrA(67) |
| CD2343_fa | senterica | 155 | aroC(10) | dnaN(60) | hemD(58) | hisD(66) | purE(6) | sucA(65) | thrA(16) |
| CD2346_fa | senterica | 23 | aroC(13) | dnaN(11) | hemD(16) | hisD(15) | purE(12) | sucA(15) | thrA(4) |
| CD2354_fa | senterica | 11 | aroC(5) | dnaN(2) | hemD(3) | hisD(7) | purE(6) | sucA(6) | thrA(11) |
| CY1701_fna | senterica | 11 | aroC(5) | dnaN(2) | hemD(3) | hisD(7) | purE(6) | sucA(6) | thrA(11) |
| CY1715_fna | senterica | 11 | aroC(5) | dnaN(2) | hemD(3) | hisD(7) | purE(6) | sucA(6) | thrA(11) |
| CY1716_fna | senterica | 11 | aroC(5) | dnaN(2) | hemD(3) | hisD(7) | purE(6) | sucA(6) | thrA(11) |
| CY1717_fna | senterica | 11 | aroC(5) | dnaN(2) | hemD(3) | hisD(7) | purE(6) | sucA(6) | thrA(11) |
| CY1810_fa | senterica | 11 | aroC(5) | dnaN(2) | hemD(3) | hisD(7) | purE(6) | sucA(6) | thrA(11) |
| CY1839_fa | senterica | 11 | aroC(5) | dnaN(2) | hemD(3) | hisD(7) | purE(6) | sucA(6) | thrA(11) |
| CY1880_fa | senterica | 11 | aroC(5) | dnaN(2) | hemD(3) | hisD(7) | purE(6) | sucA(6) | thrA(11) |
| CY1891_fa | senterica | 11 | aroC(5) | dnaN(2) | hemD(3) | hisD(7) | purE(6) | sucA(6) | thrA(11) |
| CY18925_fna | senterica | 11 | aroC(5) | dnaN(2) | hemD(3) | hisD(7) | purE(6) | sucA(6) | thrA(11) |
| CY1900_fna | senterica | 11 | aroC(5) | dnaN(2) | hemD(3) | hisD(7) | purE(6) | sucA(6) | thrA(11) |
| CY1925_fna | senterica | 11 | aroC(5) | dnaN(2) | hemD(3) | hisD(7) | purE(6) | sucA(6) | thrA(11) |
| CY1956_fna | senterica | 11 | aroC(5) | dnaN(2) | hemD(3) | hisD(7) | purE(6) | sucA(6) | thrA(11) |
| CY1960_fna | senterica | 11 | aroC(5) | dnaN(2) | hemD(3) | hisD(7) | purE(6) | sucA(6) | thrA(11) |
| CY1976_fna | senterica | 11 | aroC(5) | dnaN(2) | hemD(3) | hisD(7) | purE(6) | sucA(6) | thrA(11) |
| CY2002_fna | senterica | 11 | aroC(5) | dnaN(2) | hemD(3) | hisD(7) | purE(6) | sucA(6) | thrA(11) |
| CY2004_fna | senterica | 11 | aroC(5) | dnaN(2) | hemD(3) | hisD(7) | purE(6) | sucA(6) | thrA(11) |
| CY2005_fna | senterica | 11 | aroC(5) | dnaN(2) | hemD(3) | hisD(7) | purE(6) | sucA(6) | thrA(11) |
| CY2010_fna | senterica | 11 | aroC(5) | dnaN(2) | hemD(3) | hisD(7) | purE(6) | sucA(6) | thrA(11) |
| CY2020_fna | senterica | 11 | aroC(5) | dnaN(2) | hemD(3) | hisD(7) | purE(6) | sucA(6) | thrA(11) |
| CY2022_fna | senterica | 11 | aroC(5) | dnaN(2) | hemD(3) | hisD(7) | purE(6) | sucA(6) | thrA(11) |
| CY2023_fna | senterica | 11 | aroC(5) | dnaN(2) | hemD(3) | hisD(7) | purE(6) | sucA(6) | thrA(11) |
| CY2025_fna | senterica | 11 | aroC(5) | dnaN(2) | hemD(3) | hisD(7) | purE(6) | sucA(6) | thrA(11) |
| CY2026_fna | senterica | 11 | aroC(5) | dnaN(2) | hemD(3) | hisD(7) | purE(6) | sucA(6) | thrA(11) |
| CY2027_fna | senterica | - | aroC(5) | dnaN(-) | hemD(-) | hisD(7) | purE(6) | sucA(6) | thrA(11) |
| CY2030_fna | senterica | 11 | aroC(5) | dnaN(2) | hemD(3) | hisD(7) | purE(6) | sucA(6) | thrA(11) |
| CY2039_fna | senterica | 11 | aroC(5) | dnaN(2) | hemD(3) | hisD(7) | purE(6) | sucA(6) | thrA(11) |
| CY204_fna | senterica | 11 | aroC(5) | dnaN(2) | hemD(3) | hisD(7) | purE(6) | sucA(6) | thrA(11) |
| CY2043_fna | senterica | 11 | aroC(5) | dnaN(2) | hemD(3) | hisD(7) | purE(6) | sucA(6) | thrA(11) |
| CY2063_fna | senterica | 11 | aroC(5) | dnaN(2) | hemD(3) | hisD(7) | purE(6) | sucA(6) | thrA(11) |
| CY2065_fna | senterica | 11 | aroC(5) | dnaN(2) | hemD(3) | hisD(7) | purE(6) | sucA(6) | thrA(11) |
| CY2068_fna | senterica | 11 | aroC(5) | dnaN(2) | hemD(3) | hisD(7) | purE(6) | sucA(6) | thrA(11) |
| CY2070_fna | senterica | 11 | aroC(5) | dnaN(2) | hemD(3) | hisD(7) | purE(6) | sucA(6) | thrA(11) |
| CY2071_fna | senterica | 11 | aroC(5) | dnaN(2) | hemD(3) | hisD(7) | purE(6) | sucA(6) | thrA(11) |
| CY2074_fna | senterica | 11 | aroC(5) | dnaN(2) | hemD(3) | hisD(7) | purE(6) | sucA(6) | thrA(11) |
| CY2084_fna | senterica | 11 | aroC(5) | dnaN(2) | hemD(3) | hisD(7) | purE(6) | sucA(6) | thrA(11) |
| CY2085_fna | senterica | 11 | aroC(5) | dnaN(2) | hemD(3) | hisD(7) | purE(6) | sucA(6) | thrA(11) |
| CY2091_fna | senterica | 11 | aroC(5) | dnaN(2) | hemD(3) | hisD(7) | purE(6) | sucA(6) | thrA(11) |
| CY2097_fna | senterica | 11 | aroC(5) | dnaN(2) | hemD(3) | hisD(7) | purE(6) | sucA(6) | thrA(11) |
| CY2101_fna | senterica | 11 | aroC(5) | dnaN(2) | hemD(3) | hisD(7) | purE(6) | sucA(6) | thrA(11) |
| CY2201_fa | senterica | 307 | aroC(2) | dnaN(14) | hemD(24) | hisD(14) | purE(2) | sucA(19) | thrA(107) |
| CY2204_fa | senterica | 11 | aroC(5) | dnaN(2) | hemD(3) | hisD(7) | purE(6) | sucA(6) | thrA(11) |
| CY2208_fa | senterica | 11 | aroC(5) | dnaN(2) | hemD(3) | hisD(7) | purE(6) | sucA(6) | thrA(11) |
| CY2211_fa | senterica | 11 | aroC(5) | dnaN(2) | hemD(3) | hisD(7) | purE(6) | sucA(6) | thrA(11) |
| CY4_fna | senterica | 11 | aroC(5) | dnaN(2) | hemD(3) | hisD(7) | purE(6) | sucA(6) | thrA(11) |
| SSH1603_fna | senterica | 34 | aroC(10) | dnaN(19) | hemD(12) | hisD(9) | purE(5) | sucA(9) | thrA(2) |
| SSH1703_fna | senterica | 34 | aroC(10) | dnaN(19) | hemD(12) | hisD(9) | purE(5) | sucA(9) | thrA(2) |
| SSH17032_fna | senterica | 34 | aroC(10) | dnaN(19) | hemD(12) | hisD(9) | purE(5) | sucA(9) | thrA(2) |
| SSH17035_fna | senterica | 34 | aroC(10) | dnaN(19) | hemD(12) | hisD(9) | purE(5) | sucA(9) | thrA(2) |
| SSH1704_fna | senterica | 19 | aroC(10) | dnaN(7) | hemD(12) | hisD(9) | purE(5) | sucA(9) | thrA(2) |
| SSH17042_fna | senterica | 34 | aroC(10) | dnaN(19) | hemD(12) | hisD(9) | purE(5) | sucA(9) | thrA(2) |
| SSH17043_fna | senterica | 34 | aroC(10) | dnaN(19) | hemD(12) | hisD(9) | purE(5) | sucA(9) | thrA(2) |
| SSH1710_fna | senterica | 34 | aroC(10) | dnaN(19) | hemD(12) | hisD(9) | purE(5) | sucA(9) | thrA(2) |
| SSH1719_fna | senterica | 34 | aroC(10) | dnaN(19) | hemD(12) | hisD(9) | purE(5) | sucA(9) | thrA(2) |
| SSH1726_fna | senterica | 34 | aroC(10) | dnaN(19) | hemD(12) | hisD(9) | purE(5) | sucA(9) | thrA(2) |
| SSH1730_fna | senterica | 34 | aroC(10) | dnaN(19) | hemD(12) | hisD(9) | purE(5) | sucA(9) | thrA(2) |
| SSH1795_fna | senterica | 19 | aroC(10) | dnaN(7) | hemD(12) | hisD(9) | purE(5) | sucA(9) | thrA(2) |
| SSH1805_fna | senterica | 34 | aroC(10) | dnaN(19) | hemD(12) | hisD(9) | purE(5) | sucA(9) | thrA(2) |
| SSH1806_fna | senterica | 34 | aroC(10) | dnaN(19) | hemD(12) | hisD(9) | purE(5) | sucA(9) | thrA(2) |
| SSH1809_fna | senterica | 34 | aroC(10) | dnaN(19) | hemD(12) | hisD(9) | purE(5) | sucA(9) | thrA(2) |
| SSH18104_fna | senterica | 34 | aroC(10) | dnaN(19) | hemD(12) | hisD(9) | purE(5) | sucA(9) | thrA(2) |
| SSH1813_fna | senterica | 19 | aroC(10) | dnaN(7) | hemD(12) | hisD(9) | purE(5) | sucA(9) | thrA(2) |
| SSH1817_fa | senterica | 19 | aroC(10) | dnaN(7) | hemD(12) | hisD(9) | purE(5) | sucA(9) | thrA(2) |
| SSH1825_fna | senterica | 34 | aroC(10) | dnaN(19) | hemD(12) | hisD(9) | purE(5) | sucA(9) | thrA(2) |
| SSH18251_fna | senterica | 34 | aroC(10) | dnaN(19) | hemD(12) | hisD(9) | purE(5) | sucA(9) | thrA(2) |
| SSH1826_fna | senterica | 34 | aroC(10) | dnaN(19) | hemD(12) | hisD(9) | purE(5) | sucA(9) | thrA(2) |
| SSH18542_fna | senterica | 19 | aroC(10) | dnaN(7) | hemD(12) | hisD(9) | purE(5) | sucA(9) | thrA(2) |
| SSH18547_fna | senterica | 34 | aroC(10) | dnaN(19) | hemD(12) | hisD(9) | purE(5) | sucA(9) | thrA(2) |
| SSH1873_fna | senterica | 34 | aroC(10) | dnaN(19) | hemD(12) | hisD(9) | purE(5) | sucA(9) | thrA(2) |
| SSH1875_fna | senterica | 19 | aroC(10) | dnaN(7) | hemD(12) | hisD(9) | purE(5) | sucA(9) | thrA(2) |
| SSH1878_fna | senterica | 19 | aroC(10) | dnaN(7) | hemD(12) | hisD(9) | purE(5) | sucA(9) | thrA(2) |
| SSH1885_fna | senterica | 34 | aroC(10) | dnaN(19) | hemD(12) | hisD(9) | purE(5) | sucA(9) | thrA(2) |
| SSH1887_fna | senterica | 34 | aroC(10) | dnaN(19) | hemD(12) | hisD(9) | purE(5) | sucA(9) | thrA(2) |
| SSH1902_fna | senterica | 34 | aroC(10) | dnaN(19) | hemD(12) | hisD(9) | purE(5) | sucA(9) | thrA(2) |
| SSH1903_fna | senterica | 34 | aroC(10) | dnaN(19) | hemD(12) | hisD(9) | purE(5) | sucA(9) | thrA(2) |
| SSH1915_fna | senterica | 34 | aroC(10) | dnaN(19) | hemD(12) | hisD(9) | purE(5) | sucA(9) | thrA(2) |
| SSH1916_fna | senterica | 34 | aroC(10) | dnaN(19) | hemD(12) | hisD(9) | purE(5) | sucA(9) | thrA(2) |
| SSH1924_fna | senterica | 19 | aroC(10) | dnaN(7) | hemD(12) | hisD(9) | purE(5) | sucA(9) | thrA(2) |
| SSH1927_fna | senterica | 34 | aroC(10) | dnaN(19) | hemD(12) | hisD(9) | purE(5) | sucA(9) | thrA(2) |
| SSH1934_fna | senterica | 19 | aroC(10) | dnaN(7) | hemD(12) | hisD(9) | purE(5) | sucA(9) | thrA(2) |
| SSH1935_fa | senterica | 19 | aroC(10) | dnaN(7) | hemD(12) | hisD(9) | purE(5) | sucA(9) | thrA(2) |
| SSH1936_fna | senterica | 34 | aroC(10) | dnaN(19) | hemD(12) | hisD(9) | purE(5) | sucA(9) | thrA(2) |
| SSH1947_fna | senterica | 34 | aroC(10) | dnaN(19) | hemD(12) | hisD(9) | purE(5) | sucA(9) | thrA(2) |
| SSH1948_fna | senterica | 34 | aroC(10) | dnaN(19) | hemD(12) | hisD(9) | purE(5) | sucA(9) | thrA(2) |
| SSH1961_fna | senterica | 34 | aroC(10) | dnaN(19) | hemD(12) | hisD(9) | purE(5) | sucA(9) | thrA(2) |
| SSH1962_fna | senterica | 34 | aroC(10) | dnaN(19) | hemD(12) | hisD(9) | purE(5) | sucA(9) | thrA(2) |
| SSH1963_fna | senterica | 34 | aroC(10) | dnaN(19) | hemD(12) | hisD(9) | purE(5) | sucA(9) | thrA(2) |
| SSH1965_fna | senterica | 34 | aroC(10) | dnaN(19) | hemD(12) | hisD(9) | purE(5) | sucA(9) | thrA(2) |
| SSH1966_fna | senterica | 34 | aroC(10) | dnaN(19) | hemD(12) | hisD(9) | purE(5) | sucA(9) | thrA(2) |
| SSH1967_fna | senterica | 19 | aroC(10) | dnaN(7) | hemD(12) | hisD(9) | purE(5) | sucA(9) | thrA(2) |
| SSH2009_fna | senterica | 34 | aroC(10) | dnaN(19) | hemD(12) | hisD(9) | purE(5) | sucA(9) | thrA(2) |
| SSH20091_fna | senterica | 34 | aroC(10) | dnaN(19) | hemD(12) | hisD(9) | purE(5) | sucA(9) | thrA(2) |
| SSH20092_fna | senterica | 34 | aroC(10) | dnaN(19) | hemD(12) | hisD(9) | purE(5) | sucA(9) | thrA(2) |
| SSH2015_fna | senterica | 34 | aroC(10) | dnaN(19) | hemD(12) | hisD(9) | purE(5) | sucA(9) | thrA(2) |
| SSH2019_fna | senterica | 19 | aroC(10) | dnaN(7) | hemD(12) | hisD(9) | purE(5) | sucA(9) | thrA(2) |
| SSH2021_fna | senterica | 34 | aroC(10) | dnaN(19) | hemD(12) | hisD(9) | purE(5) | sucA(9) | thrA(2) |
| SSH2028_fna | senterica | 34 | aroC(10) | dnaN(19) | hemD(12) | hisD(9) | purE(5) | sucA(9) | thrA(2) |
| SSH2029_fna | senterica | 34 | aroC(10) | dnaN(19) | hemD(12) | hisD(9) | purE(5) | sucA(9) | thrA(2) |
| SSH2035_fna | senterica | 34 | aroC(10) | dnaN(19) | hemD(12) | hisD(9) | purE(5) | sucA(9) | thrA(2) |
| SSH2038_fna | senterica | 34 | aroC(10) | dnaN(19) | hemD(12) | hisD(9) | purE(5) | sucA(9) | thrA(2) |
| SSH2040_fna | senterica | 19 | aroC(10) | dnaN(7) | hemD(12) | hisD(9) | purE(5) | sucA(9) | thrA(2) |
| SSH2042_fna | senterica | 19 | aroC(10) | dnaN(7) | hemD(12) | hisD(9) | purE(5) | sucA(9) | thrA(2) |
| SSH2053_fna | senterica | 19 | aroC(10) | dnaN(7) | hemD(12) | hisD(9) | purE(5) | sucA(9) | thrA(2) |
| SSH2055_fna | senterica | 34 | aroC(10) | dnaN(19) | hemD(12) | hisD(9) | purE(5) | sucA(9) | thrA(2) |
| SSH2057_fna | senterica | 34 | aroC(10) | dnaN(19) | hemD(12) | hisD(9) | purE(5) | sucA(9) | thrA(2) |
| SSH2062_fna | senterica | 34 | aroC(10) | dnaN(19) | hemD(12) | hisD(9) | purE(5) | sucA(9) | thrA(2) |
| SSH2067_fna | senterica | 34 | aroC(10) | dnaN(19) | hemD(12) | hisD(9) | purE(5) | sucA(9) | thrA(2) |
| SSH2076_fna | senterica | 34 | aroC(10) | dnaN(19) | hemD(12) | hisD(9) | purE(5) | sucA(9) | thrA(2) |
| SSH2078_fna | senterica | 19 | aroC(10) | dnaN(7) | hemD(12) | hisD(9) | purE(5) | sucA(9) | thrA(2) |
| SSH2093_fna | senterica | 34 | aroC(10) | dnaN(19) | hemD(12) | hisD(9) | purE(5) | sucA(9) | thrA(2) |
| SSH2099_fna | senterica | - | aroC(10) | dnaN(19) | hemD(12) | hisD(9) | purE(~5) | sucA(9) | thrA(2) |
| SSH2128_fna | senterica | 34 | aroC(10) | dnaN(19) | hemD(12) | hisD(9) | purE(5) | sucA(9) | thrA(2) |

**Table S2. Serotyping results of *Salmonella* isolates**

| Sample | serovar | antigenic_formula | serogroup |
| --- | --- | --- | --- |
| SSH20092_fna | I 1,4,[5],12:i:- | 1,4,[5],12:i:- | B |
| SSH20091_fna | I 1,4,[5],12:i:- | 1,4,[5],12:i:- | B |
| SSH18547_fna | I 1,4,[5],12:i:- | 1,4,[5],12:i:- | B |
| SSH18542_fna | Typhimurium | 1,4,[5],12:i:1,2 | B |
| SSH18251_fna | I 1,4,[5],12:i:- | 1,4,[5],12:i:- | B |
| SSH18104_fna | I 1,4,[5],12:i:- | 1,4,[5],12:i:- | B |
| SSH17043_fna | I 1,4,[5],12:i:- | 1,4,[5],12:i:- | B |
| SSH17042_fna | I 1,4,[5],12:i:- | 1,4,[5],12:i:- | B |
| SSH17035_fna | I 1,4,[5],12:i:- | 1,4,[5],12:i:- | B |
| SSH17032_fna | I 1,4,[5],12:i:- | 1,4,[5],12:i:- | B |
| SSH2128_fna | I 1,4,[5],12:i:- | 1,4,[5],12:i:- | B |
| SSH2099_fna | I 1,4,[5],12:i:- | 1,4,[5],12:i:- | B |
| SSH2093_fna | I 1,4,[5],12:i:- | 1,4,[5],12:i:- | B |
| SSH2078_fna | Typhimurium | 1,4,[5],12:i:1,2 | B |
| SSH2076_fna | I 1,4,[5],12:i:- | 1,4,[5],12:i:- | B |
| SSH2067_fna | I 1,4,[5],12:i:- | 1,4,[5],12:i:- | B |
| SSH2062_fna | I 1,4,[5],12:i:- | 1,4,[5],12:i:- | B |
| SSH2057_fna | I 1,4,[5],12:i:- | 1,4,[5],12:i:- | B |
| SSH2055_fna | I 1,4,[5],12:i:- | 1,4,[5],12:i:- | B |
| SSH2053_fna | Typhimurium | 1,4,[5],12:i:1,2 | B |
| SSH2042_fna | Typhimurium | 1,4,[5],12:i:1,2 | B |
| SSH2040_fna | Typhimurium | 1,4,[5],12:i:1,2 | B |
| SSH2038_fna | I 1,4,[5],12:i:- | 1,4,[5],12:i:- | B |
| SSH2035_fna | I 1,4,[5],12:i:- | 1,4,[5],12:i:- | B |
| SSH2029_fna | I 1,4,[5],12:i:- | 1,4,[5],12:i:- | B |
| SSH2028_fna | I 1,4,[5],12:i:- | 1,4,[5],12:i:- | B |
| SSH2021_fna | I 1,4,[5],12:i:- | 1,4,[5],12:i:- | B |
| SSH2019_fna | Typhimurium | 1,4,[5],12:i:1,2 | B |
| SSH2015_fna | I 1,4,[5],12:i:- | 1,4,[5],12:i:- | B |
| SSH2009_fna | I 1,4,[5],12:i:- | 1,4,[5],12:i:- | B |
| SSH1967_fna | Typhimurium | 1,4,[5],12:i:1,2 | B |
| SSH1966_fna | I 1,4,[5],12:i:- | 1,4,[5],12:i:- | B |
| SSH1965_fna | I 1,4,[5],12:i:- | 1,4,[5],12:i:- | B |
| SSH1963_fna | I 1,4,[5],12:i:- | 1,4,[5],12:i:- | B |
| SSH1962_fna | I 1,4,[5],12:i:- | 1,4,[5],12:i:- | B |
| SSH1961_fna | I 1,4,[5],12:i:- | 1,4,[5],12:i:- | B |
| SSH1948_fna | Typhimurium | 1,4,[5],12:i:1,2 | B |
| SSH1947_fna | I 1,4,[5],12:i:- | 1,4,[5],12:i:- | B |
| SSH1936_fna | I 1,4,[5],12:i:- | 1,4,[5],12:i:- | B |
| SSH1935_fa | Typhimurium | 1,4,[5],12:i:1,2 | B |
| SSH1934_fna | Typhimurium | 1,4,[5],12:i:1,2 | B |
| SSH1927_fna | I 1,4,[5],12:i:- | 1,4,[5],12:i:- | B |
| SSH1924_fna | Typhimurium | 1,4,[5],12:i:1,2 | B |
| SSH1916_fna | I 1,4,[5],12:i:- | 1,4,[5],12:i:- | B |
| SSH1915_fna | I 1,4,[5],12:i:- | 1,4,[5],12:i:- | B |
| SSH1903_fna | I 1,4,[5],12:i:- | 1,4,[5],12:i:- | B |
| SSH1902_fna | I 1,4,[5],12:i:- | 1,4,[5],12:i:- | B |
| SSH1887_fna | I 1,4,[5],12:i:- | 1,4,[5],12:i:- | B |
| SSH1885_fna | I 1,4,[5],12:i:- | 1,4,[5],12:i:- | B |
| SSH1878_fna | Typhimurium | 1,4,[5],12:i:1,2 | B |
| SSH1875_fna | Typhimurium | 1,4,[5],12:i:1,2 | B |
| SSH1873_fna | I 1,4,[5],12:i:- | 1,4,[5],12:i:- | B |
| SSH1826_fna | I 1,4,[5],12:i:- | 1,4,[5],12:i:- | B |
| SSH1825_fna | I 1,4,[5],12:i:- | 1,4,[5],12:i:- | B |
| SSH1817_fa | Typhimurium | 1,4,[5],12:i:1,2 | B |
| SSH1813_fna | Typhimurium | 1,4,[5],12:i:1,2 | B |
| SSH1809_fna | I 1,4,[5],12:i:- | 1,4,[5],12:i:- | B |
| SSH1806_fna | I 1,4,[5],12:i:- | 1,4,[5],12:i:- | B |
| SSH1805_fna | I 1,4,[5],12:i:- | 1,4,[5],12:i:- | B |
| SSH1795_fna | Typhimurium | 1,4,[5],12:i:1,2 | B |
| SSH1730_fna | I 1,4,[5],12:i:- | 1,4,[5],12:i:- | B |
| SSH1726_fna | I 1,4,[5],12:i:- | 1,4,[5],12:i:- | B |
| SSH1719_fna | I 1,4,[5],12:i:- | 1,4,[5],12:i:- | B |
| SSH1710_fna | I 1,4,[5],12:i:- | 1,4,[5],12:i:- | B |
| SSH1704_fna | Typhimurium | 1,4,[5],12:i:1,2 | B |
| SSH1703_fna | I 1,4,[5],12:i:- | 1,4,[5],12:i:- | B |
| SSH1603_fna | I 1,4,[5],12:i:- | 1,4,[5],12:i:- | B |
| CY18925_fna | Enteritidis | 1,9,12:g,m:- | D1 |
| CY2211_fa | Enteritidis | 1,9,12:g,m:- | D1 |
| CY2208_fa | Enteritidis | 1,9,12:g,m:- | D1 |
| CY2204_fa | Enteritidis | 1,9,12:g,m:- | D1 |
| CY2201_fa | Paratyphi B var. Java | 1,4,[5],12:b:1,2 | B |
| CY2101_fna | Enteritidis | 1,9,12:g,m:- | D1 |
| CY2097_fna | Enteritidis | 1,9,12:g,m:- | D1 |
| CY2091_fna | Enteritidis | 1,9,12:g,m:- | D1 |
| CY2085_fna | Enteritidis | 1,9,12:g,m:- | D1 |
| CY2084_fna | Enteritidis | 1,9,12:g,m:- | D1 |
| CY2074_fna | Enteritidis | 1,9,12:g,m:- | D1 |
| CY2071_fna | Enteritidis | 1,9,12:g,m:- | D1 |
| CY2070_fna | Enteritidis | 1,9,12:g,m:- | D1 |
| CY2068_fna | Enteritidis | 1,9,12:g,m:- | D1 |
| CY2065_fna | Enteritidis | 1,9,12:g,m:- | D1 |
| CY2063_fna | Enteritidis | 1,9,12:g,m:- | D1 |
| CY2043_fna | Enteritidis | 1,9,12:g,m:- | D1 |
| CY2039_fna | Enteritidis | 1,9,12:g,m:- | D1 |
| CY2030_fna | Enteritidis | 1,9,12:g,m:- | D1 |
| CY2027_fna | Enteritidis | 1,9,12:g,m:- | D1 |
| CY2026_fna | Enteritidis | 1,9,12:g,m:- | D1 |
| CY2025_fna | Enteritidis | 1,9,12:g,m:- | D1 |
| CY2023_fna | Enteritidis | 1,9,12:g,m:- | D1 |
| CY2022_fna | Enteritidis | 1,9,12:g,m:- | D1 |
| CY2020_fna | Enteritidis | 1,9,12:g,m:- | D1 |
| CY2010_fna | Enteritidis | 1,9,12:g,m:- | D1 |
| CY2005_fna | Enteritidis | 1,9,12:g,m:- | D1 |
| CY2004_fna | Enteritidis | 1,9,12:g,m:- | D1 |
| CY2002_fna | Enteritidis | 1,9,12:g,m:- | D1 |
| CY1976_fna | Enteritidis | 1,9,12:g,m:- | D1 |
| CY1960_fna | Enteritidis | 1,9,12:g,m:- | D1 |
| CY1956_fna | Enteritidis | 1,9,12:g,m:- | D1 |
| CY1925_fna | Enteritidis | 1,9,12:g,m:- | D1 |
| CY1900_fna | Enteritidis | 1,9,12:g,m:- | D1 |
| CY1891_fa | Enteritidis | 1,9,12:g,m:- | D1 |
| CY1880_fa | Enteritidis | 1,9,12:g,m:- | D1 |
| CY1839_fa | Enteritidis | 1,9,12:g,m:- | D1 |
| CY1810_fa | Enteritidis | 1,9,12:g,m:- | D1 |
| CY1717_fna | Enteritidis | 1,9,12:g,m:- | D1 |
| CY1716_fna | Enteritidis | 1,9,12:g,m:- | D1 |
| CY1715_fna | Enteritidis | 1,9,12:g,m:- | D1 |
| CY1701_fna | Enteritidis | 1,9,12:g,m:- | D1 |
| CY204_fna | Enteritidis | 1,9,12:g,m:- | D1 |
| CY4_fna | Enteritidis | 1,9,12:g,m:- | D1 |
| CD2354_fa | Enteritidis | 1,9,12:g,m:- | D1 |
| CD2346_fa | Oranienburg | 6,7,14:m,t:- | C1 |
| CD2343_fa | London | 3,{10}{15}:l,v:1,6 | E1 |
| CD2332_fa | Kentucky | 8,20:i:z6 | C2-C3 |
| CD2330_fa | Saintpaul | 1,4,[5],12:e,h:1,2 | B |
| CD2325_fa | Enteritidis | 1,9,12:g,m:- | D1 |
| CD2324_fa | Senftenberg | 1,3,19:g,[s],t:- | E1 |
| CD2321_fa | London | 3,{10}{15}:l,v:1,6 | E1 |
| CD2313_fa | Saintpaul | 1,4,[5],12:e,h:1,2 | B |
| CD2310_fa | Anatum | 3,{10}{15}{15,34}:e,h:1,6 | E1 |
| CD2308_fa | Goldcoast | 6,8:r:l,w | C2-C3 |
| CD2302_fa | Kentucky | 8,20:i:z6 | C2-C3 |
| CD2287_fa | London | 3,{10}{15}:l,v:1,6 | E1 |
| CD2286_fa | Agona | 1,4,[5],12:f,g,s:- | B |
| CD2281_fa | Enteritidis | 1,9,12:g,m:- | D1 |
| CD2277_fa | Goldcoast | 6,8:r:l,w | C2-C3 |
| CD2254_fa | Goldcoast | 6,8:r:l,w | C2-C3 |
| CD2243_fa | Rissen | 6,7,14:f,g:- | F |
| CD2241_fa | Rissen | 6,7,14:f,g:- | F |
| CD2231_fa | Amsterdam | 3,{10}{15}{15,34}:g,m,s:- | E1 |
| CD2230_fa | Goldcoast | 6,8:r:l,w | C2-C3 |
| CD2207_fa | Enteritidis | 1,9,12:g,m:- | D1 |
| CD2196_fa | Liverpool | 1,3,19:d:e,n,z15 | E1 |
| CD2190_fa | Braenderup | 6,7,14:e,h:e,n,z15 | C1 |
| CD2187_fa | London | 3,{10}{15}:l,v:1,6 | E1 |
| CD2186_fa | Agona | 1,4,[5],12:f,g,s:- | B |
| CD2184_fa | Agona | 1,4,[5],12:f,g,s:- | B |
| CD2176_fa | Rissen | 6,7,14:f,g:- | F |
| CD2133_fa | London | 3,{10}{15}:l,v:1,6 | E1 |
| CD2122_fa | Derby | 1,4,[5],12:f,g:- | B |
| CD2114_fa | Mbandaka | 6,7,14:z10:e,n,z15 | F |
| CD2111_fa | Goldcoast | 6,8:r:l,w | C2-C3 |
| CD2105_fa | Infantis | 6,7,14:r:1,5 | C1 |
| CD2064_fa | Derby | 1,4,[5],12:f,g:- | B |
| CD2061_fa | Reading | 1,4,[5],12:e,h:1,5 | B |
| CD2060_fa | Litchfield | 6,8:l,v:1,2 | C2-C3 |
| CD2059_fa | Agona | 1,4,[5],12:f,g,s:- | B |
| CD2050_fa | Braenderup | 6,7,14:e,h:e,n,z15 | C1 |
| CD2045_fa | London | 3,{10}{15}:l,v:1,6 | E1 |
| CD2032_fa | Agona | 1,4,[5],12:f,g,s:- | B |
| CD2018_fa | London | 3,{10}{15}:l,v:1,6 | E1 |
| CD2014_fa | Schwarzengrund | 1,4,12,27:d:1,7 | B |
| CD2008_fa | London | 3,{10}{15}:l,v:1,6 | E1 |
| CD2001_fa | Paratyphi B var. Java | 1,4,[5],12:b:1,2 | B |
| CD1970_fa | Thompson | 6,7,14:k:1,5 | C1 |
| CD1969_fa | London | 3,{10}{15}:l,v:1,6 | E1 |
| CD1959_fa | Indiana | 1,4,12:z:1,7 | B |
| CD1950_fa | London | 3,{10}{15}:l,v:1,6 | E1 |
| CD1949_fa | London | 3,{10}{15}:l,v:1,6 | E1 |
| CD1939_fa | Give | 3,{10}{15}{15,34}:l,v:1,7 | E1 |
| CD1932_fa | Rissen | 6,7,14:f,g:- | F |
| CD1920_fa | London | 3,{10}{15}:l,v:1,6 | E1 |
| CD1918_fa | Kottbus | 6,8:e,h:1,5 | C2-C3 |
| CD1909_fa | I 1,4,[5],12:i:- | 1,4,[5],12:i:- | B |
| CD1904_fa | I 1,4,[5],12:i:- | 1,4,[5],12:i:- | B |
| CD1902_fa | I 1,4,[5],12:i:- | 1,4,[5],12:i:- | B |
| CD1901_fa | Thompson | 6,7,14:k:1,5 | C1 |
| CD1890_fa | Livingstone | 6,7,14:d:l,w | F |
| CD1889_fa | Mbandaka | 6,7,14:z10:e,n,z15 | F |
| CD1888_fa | Stanley | 1,4,[5],12,[27]:d:1,2 | B |
| CD1886_fa | Stanley | 1,4,[5],12,[27]:d:1,2 | B |
| CD1884_fa | Mbandaka | 6,7,14:z10:e,n,z15 | F |
| CD1883_fa | Livingstone | 6,7,14:d:l,w | C1 |
| CD1882_fa | London | 3,{10}{15}:l,v:1,6 | E1 |
| CD1881_fa | Livingstone | 6,7,14:d:l,w | C1 |
| CD1863_fa | Agona | 1,4,[5],12:f,g,s:- | B |
| CD1844_fa | Mbandaka | 6,7,14:z10:e,n,z15 | F |
| CD1822_fa | Goldcoast | 6,8:r:l,w | C2-C3 |
| CD1821_fa | London | 3,{10}{15}:l,v:1,6 | E1 |
| CD1819_fa | Mbandaka | 6,7,14:z10:e,n,z15 | F |
| CD1816_fa | Souza\|Madjorio | -:d:e,n,z15 | E1 |
| CD1815_fa | Meleagridis | 3,{10}{15}{15,34}:e,h:l,w | E1 |
| CD1814_fa | Meleagridis | 3,{10}{15}{15,34}:e,h:l,w | E1 |
| CD1812_fa | Give | 3,{10}{15}{15,34}:l,v:1,7 | E1 |

**Table S3. Historical phenotypic antimicrobial resistance profiles of prevalent *Salmonella* serovars in the Jinan region**

| **Serovar** | **No. of Isolates** | **Resistance Profile (Core)** | **Resistance Profile (Variable)** | **Key Observations / Correlation with WGS Data** |
| --- | --- | --- | --- | --- |
| **Goldcoast** | 7 | AMP, TET, CHL, SXT, STR | CIP (High), CAZ, CTX | Represents a classic MDR phenotype. The consistent resistance to AMP, TET, SXT, and STR strongly correlates with the high prevalence of corresponding genes (blaTEM-1B, tetA, sul, aadA) identified by WGS. High-level CIP resistance suggests accompanying QRDR mutations. |
| **London** | 16 | TET, STR | AMP, CHL, SXT, CIP, CTX | Exhibits a diverse resistance spectrum. Universal resistance to TET and STR aligns with the near-ubiquitous presence of tetA and aadA genes. Variable resistance to other antibiotics indicates genetic plasticity in acquiring additional resistance determinants. |
| **Infantis** | 5 | AMP, TET, CHL, SXT, STR | CIP (Variable), CTX | Displays a stable MDR core. The phenotype perfectly matches the expected genotype (blaTEM, tetA, sul, aadA), providing strong evidence for a consistent genotype-phenotype relationship in this serovar. |
| **Stanley** | 5 | AMP, TET, CHL, SXT, STR | CIP (Variable) | Characterized by a conserved MDR core. Resistance to AMP, TET, etc., is consistent with the detection of relevant genes. Variable CIP resistance underscores the complex genotype-phenotype relationship for fluoroquinolones. |
| **Agona** | 6 | STR | AMP, TET, SXT | Demonstrates a more constrained resistance profile in some isolates. Universal STR resistance correlates with the common aadA genes. Susceptibility to AMP and TET in some isolates suggests the absence of blaTEM-1B or tetA, highlighting population diversity. |
| **Enteritidis** | (From Current Study) | (Genotypic: TET, NAL, AMP) |  | The dominant ST11/Enteritidis in our WGS data carries tetA, blaTEM-1B, and QRDR mutations. Historical phenotypes for this serovar commonly show congruent resistance to TET, AMP, and NAL, supporting our genotypic findings. |
| **I 4,[5],12:i:-** | (From Current Study) | (Genotypic: MDR) |  | The epidemic ST34/I 4,[5],12:i:- clone identified by WGS exhibits an MDR genotype. Historical data consistently shows high MDR rates in this serovar, robustly corroborating its role as a major vehicle for MDR in the local context. |
